# Exploring and modeling the reading-writing connection in EFL integrated writing

**DOI:** 10.3389/fpsyg.2023.1161272

**Published:** 2023-07-05

**Authors:** Wei Ye, Jianda Liu

**Affiliations:** ^1^School of Foregin Languages, Guangdong Polytechnic Normal University, Guangzhou, China; ^2^Center for Linguistics and Applied Linguistics, Guangdong University of Foreign Studies, Guangzhou, China

**Keywords:** integrated writing, EFL learning, mixed-methods, grounded theory, confirmatory factor analysis

## Abstract

Reading-to-write tasks have increasingly been used in high-stakes language tests worldwide; however, the nature of the reading-writing connection is not well understood. This study utilized a mixed-methods approach to ground descriptions of EFL cognitive processes and identify process interaction patterns to determine how reading and writing were connected. Grounded theory analysis of fourteen EFL learners’ writing think-aloud protocols showed that students engaged in an interactive composing process involving source reading, comprehension monitoring, planning, language monitoring, narration monitoring, and continuity evaluation. We also conducted a confirmatory factor model study on 486 EFL learners’ responses to a self-developed writing questionnaire, which covered five factors, including reading monitoring, narration monitoring, ideational planning, continuity evaluation, and skill integration. The findings showed that reading monitoring was the only factor that had a direct and significant impact on skill integration, a composite factor covering discourse synthesizing and source using processes. Based on the discussion of the theoretical, empirical, and pedagogical implications of the current findings, we called for more studies to explore the use of three pillar skills—reading, writing, and language use—to support EFL integrated writing. We also suggested that test designers include explicit rating descriptor(s) for source using to evaluate reading comprehension, and instructors enhance reading instruction to improve task performance.

## 1. Introduction

The natural and close connections between reading and writing have long been acknowledged in language learning and use situations (Grabe and Zhang, 2016). The 21st century has seen an increase in the use of integrated tasks in tertiary gate-keeping tests worldwide, such as the TOEFL iBT. This poses a great challenge to EFL learners who have traditionally learned the language through individual skill use at the pre-tertiary level (Bruce and Hamp-Lyons, 2015). A clear understanding of the reading-writing connection is critical to ensure their success in integrated writing tests.

Previous research, situated in both L1 and L2 contexts, has shown that the reading-writing connection is complex (Knoch and Sitajalabhorn, 2013) and still unclear (Plakans et al., 2019). This study aimed to explore and model the cognitive processes that EFL learners undertake during the integrated writing process to provide an EFL-oriented perspective on the reading-writing connection issue. We employed a mixed-methods design to first obtain contextual descriptions of the EFL integrated writing process. Then, we applied quantitative approaches to find out how reading and writing processes interacted to achieve skill-integration, the key construction that signifies reading-writing connection in operation. This study collected EFL integrated writing data using the Story Continuation Writing Task (SCWT) (Ye and Ren, 2019, 2023; Shi et al., 2020). This is an integrated writing task developed for the National Matriculation English Test (NMET) in China, requiring writers to read a 350-word incomplete story and then supply a 150-word continuation. Hence, the findings also inform integrated writing test design and classroom instructions in the EFL context.

## 2. Review of literature

### 2.1. The reading-writing connection

Reading and writing are two important literacy skills (Cope and Kalantazis, 2000) that share a set of common knowledge and processes (Fitzgerald and Shanahan, 2000). In terms of language use, they are reciprocal communicative acts essential for meaning-making. In terms of language learning, they are mutually supportive literacy abilities (Grabe and Zhang, 2016) that native speakers learn to use together at an early stage of education. In terms of writing assessment, integrated writing not only offers learners an equal basis of ideas and logic to reduce content bias (Gebril and Plakans, 2009) but also enhances test authenticity by replicating academic situations in which students need to read and write together to consolidate content learning (Weigle, 2004).

However, reading and writing are also cognitively separate because they involve different cognitive systems and elicit unique processes (Berninger and Abbott, 2010). This leads to the problematic issue of muddied measurement, a topic of heated debate for decades (Alderson et al., 1995; Weir, 2005). The issue of the reading-writing connection is so important that it is frequently addressed by two of the four strands of integrated writing construct research (Zhu et al., 2016), namely writing process and skill impact studies. The other two strands are discourse studies and correlation studies with independent writing performance.

### 2.2. Integrated writing process studies

Process studies examine the range of knowledge, skills, and processes that support task performance (Messick, 1989; Weir, 2005) to determine whether the testing task elicits the language abilities that test developers aim to measure (Bachman and Palmer, 2010). Researchers often use the terms “process” and “strategy” to refer to purposeful activities that enable language users to complete the task (Petrić and Czárl, 2003; Abdel Latif, 2021). For the sake of clarity, this study will use the term “process” when referring to the concept.

Focusing on the skill integration dimension unique to integrated writing, Spivey and colleagues (Spivey, 1984, 1990, 1997; Spivey and King, 1989) identified three key discourse synthesis processes, namely organizing, selecting, and connecting, from the cognitive writing process in the first language (L1). Structural equation modeling studies of questionnaire responses (Yang and Plakans, 2012) confirmed the qualitative finding (Plakans, 2009a) that discourse synthesis was essential for task success. Moreover, Yang (2014) found that both discourse synthesizing and source using processes had direct and positive impacts on integrated writing performance. To encompass the central construct of integrated writing, Knoch and Sitajalabhorn (2013) proposed the inclusive concept of “skill integration” (p. 305), which covers both conceptual processes (e.g., summarizing key source ideas, selecting source ideas for writing, idea organization, and connecting source ideas with one’s own) and textual integration processes (e.g., transforming the source language).

Research on the shared processes between reading and writing has shed further light on the issue. Stein (1990) proposed one of the earliest models for reading-writing cognition by examining the shared processes in L1 reading and writing. The metacognitive planning and monitoring processes and the cognitive structuring and elaboration processes identified in the model were then validated by empirical studies conducted in the English as a foreign language (EFL) context (Ruiz-Funes, 2001) and English as a second language (ESL) integrated writing situations (Yang and Shi, 2003). Recently, Plakans et al. (2019) expanded the research scope for the shared processes in their study of the reading-writing connection using a writing-reading-writing task. Their studies showed that ESL writers engaged in word-level language use, reading/rereading, summarizing, and monitoring processes during both the reading and writing processes. In other words, basic language use, cognitive processes, and composing processes all contributed to establishing the reading-writing connection.

However, despite these efforts, little is known about how reading-only and writing-only processes contribute to skill integration. Given its significance as the key integrated writing construct that connects reading and writing (Knoch and Sitajalabhorn, 2013), we argue that a better understanding of this concept is essential for interpreting integrated writing performance. Moreover, most studies have demonstrated an L1-dominated perspective of integrated writing, thereby failing to address the specificity of the EFL writing process, as discussed in Manchón et al. (2009). Given the growing importance of integrated writing assessments in EFL contexts (e.g., Cumming et al., 2018), we believe that an EFL-oriented perspective derived from a contextual investigation of the EFL writing process would contribute to the discussion on the reading-writing connection.

### 2.3. Skill impact studies

While cognitive process studies seek evidence of reading-writing integration, skill impact studies aim to delineate the differential impacts of reading and writing.

Empirical studies have shown that the significance and magnitude of the impacts of reading and writing vary across tasks and contexts. While some studies found reading and writing were substantially correlated with integrated writing performance (Cumming et al., 2005; Shin and Ewert, 2014), others reported weak correlations between individual skills and task scores (Asención, 2008). Additionally, while some studies demonstrated that integrated writing is primarily a writing task (Watanabe, 2001; Li, 2014), others showed that reading is a better predictor (Yu, 2008).

In recent years, researchers have employed more sophisticated quantitative approaches to examine interactions among skill-oriented processes, such as reading and writing, and the skill integration process. For instance, in a study on a Chinese listening-reading-writing task, Liao et al. (2021) used structural equation modeling and found that the listening and writing abilities of L1 Chinese writers significantly influenced both task scores and the use of integrated writing strategies (*β* = 0.21 and 0.32), while reading had no impact on either performance variables. However, this study posited integrated writing strategy as a unitary factor comprised of discourse synthesizing, idea development, language monitoring, and content monitoring. The general impacts of writing need to be further explicated by examining interactions among these integrated writing processes. An example of this is a recent confirmatory factor analysis (CFA) cognitive process study on the SCWT (Ye and Ren, 2023), which is reported at the end of the next section.

### 2.4. The SCWT used in the NMET

The SCWT is an integrated writing task used in the NMET (National Matriculation English Test) to elicit evidence of integrated writing abilities. It requires writers to provide a 150-word continuation to a 350-word incomplete narrative. Currently, task performance is evaluated using a holistic scale that covers content development, language accuracy and diversity, as well as overall coherence and cohesion. This accounts for 25 points out of the total 150-point test.

In contrast to conventional integrated writing tasks that emphasize appropriate source use to indicate authorship (Du, 2019), the SCWT encourages extensive use of source material to build alignments between the EFL writing output and reading input, thereby scaffolding EFL learning (Wang and Wang, 2015).

Skill impact studies have shown that writing plays a more important role in SCWT writing. Wang and Qi (2013) examined the SCWT writing of 203 secondary students and found substantial correlations between task scores and writing (*r* = 0.68) as well as reading (*r* = 0.46). In a subsequent mock NMET test involving 1,180 NMET test-takers, Liu and Chen (2016) also reported that SCWT scores were related to both writing (0.70) and reading scores (0.44).

Process studies have demonstrated that the SCWT taps into skill integration processes, specifically in terms of discourse synthesis and source use. Based on a grounded analysis of the writer think-aloud protocols (TAPs) and instructor interviews, Ye and Ren (2019) found that the SCWT required both conceptual source use to support story development and textual source use to enhance coherence. Shi et al. (2020) collected 93 responses using a questionnaire developed based on previous research and identified nine process factors for SCWT writing, namely evaluating, connecting, monitoring, planning, source use, and organizing. Drawing upon EFL writing literature and proficiency descriptors, Ye and Ren (2023) developed a task-specific process questionnaire to investigate how language skill use affected SCWT performance. Interestingly, their factor analysis identified a secondary-order factor of “reading-to-connect,” comprising “reading and selecting” and “transformation.” Their confirmatory factor analysis (CFA) study of 470 responses demonstrated that the reading-to-connect factor mediated the impact of planning on writing, which then directly and positively affected test performance. Findings suggest that reading, discourse synthesizing, and source use are closely related and support the writing production process. The complex pattern of skill use indicates the need for further exploration of the interactions among the integrated writing processes. The SCWT is an ideal task to continue the line of research initiated by Ye and Ren (2019, 2023).

Previous studies on the SCWT were mostly quantitative in nature, utilizing questionnaires developed based on ESL findings (e.g., Shi et al., 2020) or theoretical language frameworks (e.g., Ye and Ren, 2023). In this study, we aimed to build upon the qualitative efforts initiated by Ye and Ren (2019) and present a mixed-method study on this task to contribute to the current discussion on the reading-writing connection. Specifically, we addressed two research questions (RQs):

1. What cognitive processes were EFL learners engaged in during integrated writing?
2. How did reading-and writing-oriented processes contribute to the reading-writing connection?

## 3. Methodology

### 3.1. Overall research design and rationale

This research presents a mixed-method study of SCWT writing processes to explore the reading-writing connection in an EFL context. For RQ 1, we grounded contextual descriptions of EFL cognitive processes from the writer TAPs. For RQ 2, we developed a process questionnaire based on the qualitative descriptions of the SCWT cognitive process obtained through open coding categories. We then used the confirmatory factor analysis (CFA) approach to examine interactions among the cognitive processes identified from the principal component analysis (PCA).

### 3.2. Participants

Two groups of EFL learners participated in the current study.

For the qualitative study in response to RQ 1, we recruited fourteen volunteer learners to collect TAP and retrospective interview data. All learners had started learning English no later than the age of nine and none had any overseas learning experience. Table 1 provides further information about the TAP volunteers, including their gender, age, academic background, and task experience. Notably, seven out of fourteen participants (50%) reported being familiar with the task, as they had practiced the SCWT in their English class.

**Table 1:** Participant Information.

Name	Gender	Age	Academic background	Task experience
Sandy	Female	20	Freshmen	+
Wendy	Female	19	Freshmen	+
Harley	Female	19	Freshmen	+
Yvonne	Female	19	Freshmen	+
Lay	Male	21	Sophomore	+
Ronald	Male	19	Freshmen	+
Gary	Male	19	Freshmen	+
Mike	Male	19	Freshmen	−
Shan	Male	19	Freshmen	−
Owen	Male	19	Freshmen	−
Phoebe	Female	19	Freshmen	−
Danielle	Female	19	Freshmen	−
Moses	Male	17	Secondary learner	−
Zack	Male	18	Secondary learner	−

For the quantitative study in response to RQ2, we recruited 486 freshmen non-English majors from three universities in South China to collect questionnaire data. Among them, there were 256 males (52.7%) and 230 females (47.3%). They had little knowledge about integrated writing, scoring an average of 1.67 on a 3-point scale measuring task familiarity, and had limited familiarity with the topic, scoring an average of 1.54 on a 3-point scale measuring story familiarity. Generally speaking, they represented a group of high-achieving NMET test-takers, with an average score of 123.2 out of 150. They relied mainly on their existing language proficiency to complete the integrated writing task. As the data was collected from first-year college students in the sixth week of their first semester, we assumed that there would be little change in participants’ language proficiency since the NMET. Therefore, their questionnaire responses were considered representative of NMET test-taker perspectives. It is important to note that the use of the SCWT is not limited to the secondary level (Wang et al., 2000) and task has gained increasing popularity in tertiary writing instruction (Chen and Cui, 2022). Hence, we believe that the SCWT is appropriate for eliciting integrated writing data from college students.

### 3.3. Instruments and procedures

#### 3.3.1. The SCWT task

TAP participants and respondents were asked to first complete an SCWT task before proceeding to the respective section for collecting cognitive process data.

Since the research was conducted before the SCWT test format was finalized in 2020, we used the prototype task from an early SCWT study (Ye and Ren, 2019) for data collection. The source text, provided in Appendix 1, consisted of 334 words and revolved around a man who accidentally became involved in a robbery on his way to work and subsequently ran away from the scene out of fear. Test-takers were required to continue the story, providing a logical and reasonable ending.

#### 3.3.2. TAP

Following Dörnyei (2007), we collected the writer TAPs in four steps: participants underwent a training and practice session, verbalized their cognitive writing processes during the task, received a retrospective interview, and finally answered a follow-up email for member-checking. To encourage verbalization, Chinese language was used, and no time limit was imposed. The coding scheme with 32 entries was presented in Section 4.1.

#### 3.3.3. The SCWT questionnaire

Based on the information provided, the initial SCWT process questionnaire items were developed using two principles.

1. Providing precise information about SCWT writing processes: The questionnaire aimed to gather detailed information about the specific processes involved in SCWT writing.
2. Ensuring comprehensibility for EFL learners with limited knowledge of linguistic terms: The questionnaire was designed to be easily understood by EFL learners who might not be familiar with complex linguistic terminology.

To refine the questionnaire, we conducted cognitive interviews with seven English-majoring sophomores and two Ph.D. candidates studying language testing. These interviews provided valuable insights, which were used to revise the questionnaire instructions and item wordings. For example, the item “monitor lexical use” was modified to include the specific example of spelling, making it easier for respondents to understand. Another change was made to the item related to “world knowledge” in the planning category, as interviewees found the phrase ambiguous. Based on their suggestions, the item was revised to “plan the content based on common logic.,” and participants need to rate the extent to which they planned the continuation content based on their common sense knowledge.

The initial questionnaire consisted of 32 items that asked learners to rate the frequency of process use on a five-point scale. These items were included in Appendix 2 of the research document.

We then employed the PCA method with varimax rotation on the questionnaire responses of 161 freshmen to reduce the item number. As a result, a final questionnaire was developed, consisting of 15 items that demonstrated strong (*β* ≥ 0.4) and unique associations with the identified factors. These final items can be found in Appendix 3 of the research document.

### 3.4. Data analysis

#### 3.4.1. Grounded analysis

To address RQ1, the first author employed an inductive approach for grounded theory analysis, following the methodology outlined by Corbin and Strauss (2008).

The analysis began with open coding, wherein the first author developed an initial coding scheme. Throughout this process, the emerging coding categories were constantly cross-referenced with the data being analyzed, allowing for refinement of the coding scheme.

Following the open coding phase, the researchers conducted axial coding. During this stage, they closely examined the raw data to identify empirical content categories that represented the key cognitive processes supporting SCWT performance. This step involved exploring and organizing the data to establish connections between categories.

To derive the final axial coding scheme, both authors engaged in discussions to review and refine the identified schemes. Through collaboration and agreement, they created an axial coding scheme that effectively represented the essential SCWT cognitive processes.

#### 3.4.2. The CFA study

Data screening revealed that 411 responses were complete and valid for further analysis. Descriptive statistics were computed to assess the normality assumption for the maximum likelihood (ML) method. Additionally, alpha coefficients and individual scales were reported to demonstrate the reliability level of the questionnaire.

The goodness of model fit was evaluated using a set of commonly used indices (Hu and Bentler, 1999). These indices include the Chi-square test (*χ*^2^, p>0.05), χ^2^/*df* (≤2.5), Tucker and Lewis index (TLI, ≥0.90), Bentler’s comparative fit index (CFI, ≥0.95), root mean square error of approximation (RMSEA, ≤0.06), and standardized root mean square residual (SRMR, ≤0.08). These indices were employed to determine how well the models fit the data.

To address RQ 2, four models were tested to identify the psychometric structure of the SCWT writing process. The first model, known as the construct-trait factor (CTF) model, assumed that the construct dimensions were distinguishable. The orthogonal-trait factor (OTF) model implied that the dimensions were unrelated. The unitary-trait factor (UTF) model assumed that all factors were indistinguishable. Finally, the higher-order-trait factor (HTF) model hypothesized the presence of an overarching secondary-order factor. These models were evaluated to determine which one best represented the data.

## 4. Results

### 4.1. SCWT cognitive process

A total of 32 open coding categories were identified and classified under six axial coding categories: source reading, comprehension monitoring, planning, language monitoring, narration monitoring, and continuity evaluation.

#### 4.1.1. Source Reading

Table 2 presented a range of processes utilized by learners, including underlining and scanning, in order to comprehend the source material. During the initial reading, learners attentively went through the text, aiming to identify significant cues. To ensure a comprehensive understanding of the source, many learners engaged in subsequent readings, sometimes even going through it for the third time.

**Table 2:** The axial coding of source reading.

Open coding	Writing examples
Underline key parts	*Jack*, the main character… *Park Avenue*, the location… (I have underlined and circled the key information in the source…)
Scan for details and key information	So where is the other suitcase? Where was it?
Read the source carefully	*(He) walked right into the young woman in front of him*…
Reread the source	Is he (the young man) the robber? I need to reread the source

#### 4.1.2. Comprehension monitoring

Inference-making and summarizing were reading processes utilized to monitor and improve source comprehension. Inference-making allowed learners to uncover implicit information, such as the personalities of the characters, as demonstrated in the writing sample presented in Table 3. Summarizing enabled learners to connect narrative elements identified in various parts of the story, creating a cohesive narrative. This process facilitated personal interpretations, as exemplified by the descriptors “nice” and “poor” in the writing example from Table 3.

**Table 3:** The axial coding of comprehension monitoring.

Open coding	Writing examples
Summarize the source	John was so nice that he helped a young man on his way to work…The bank got robbed, and then…John was taken as the bank robber by a woman…Out of fear, he ran away. (I think) this is a story about this poor man’s adventure…
Interpret the source based on inference-making	John ran away…without defending himself… He must be very timid.

#### 4.1.3. Planning

Overall task planning, which involved considering task requirements, and ideational planning for content construction, were categorized as planning processes. The writing examples presented in Table 4 demonstrated how learners engaged in global planning processes to align their writing with the required length and content development.

**Table 4:** The axial coding of planning.

Open coding	Writing examples
Think carefully about the task requirement	What kind of story development could meet the 150-word length requirement?
Plan the continuation globally before writing	It would be a story like this: John was scared, so he fled…but when he was caught, people realized it had been a mistake.
Plan the content during the writing	*He tried his best way to stay calm and…*And then what should he do?
Consider the writing length	Maybe I need to reconsider the plot. There is a 150-word limit.
Develop the continuation based on world knowledge	Can he outrun all these people and reach somewhere safe? Probably not!
Develop the continuation based on topical knowledge	What about an adventure story just like the spider-man movie…It probably took more than 150 words to explain the idea. Just forget about it.
Develop the story based on the source	Would John go back to the scene to find the young men? … No, he was not that brave.
Develop the continuation by inference making	He had to rely on himself… So …figure out a solution… to prove his innocence.
Select the storyline for production	What about the young man and the woman?… They were minor characters. Just mention them briefly in the last part….

For ideational planning, learners relied on inference-making, topical knowledge, and world knowledge to generate suitable content for the continuation. They selected a storyline that best fit their understanding of the source material. The writing example associated with topical knowledge use illustrated that EFL learners’ choice of content was influenced by their writing ability as well.

#### 4.1.4. Language monitoring

Participants considered language use as critical for task success. Hence, they closely attended to language accuracy, correctness, diversity, collocation, word use, and cohesive devices, as demonstrated by writing examples in Table 5.

**Table 5:** The axial coding of language monitoring.

Open coding	Writing examples
Monitor lexical use	How does the word suspect spell? S-u-s…Do not get it wrong!
Use pronouns and conjunctions to establish coherence	I have used the pronoun *he* too many times. Would this confuse the reader?
Monitor the collocation use	*In a hurry.* This is the right way to express the idea, right?
Monitor language accuracy	*Figure out a …*Solution? Explanation? Action? Solution!
Use diversified expressions, or avoid repetition	*Therefore* again? *Therefore, so?* What other words can I use? Just use so.
Use collocations or structures correctly	Is there any grammatical problem?
Use source language directly	What was he carrying at the time? A box? (Reread the source) It is a suitcase!
Reread the continuation	*John went as fast as a horse.* Good metaphor use!

Source use was also an essential part of language use. Sandy, an English major, believed that the repetition of key source concepts was acceptable, and paraphrasing would only create confusion.

#### 4.1.5. Narration monitoring

In addition to the general language use category, writers included language use features that were closely linked to narrative production, such as the use of past tense, dialogs, detailed descriptions, and narrative structure (as shown in Table 6), within the narration monitoring category. This classification aimed to emphasize the genre-specific writing requirements of the task. Therefore, the process of “keeping to the past tense,” which is a crucial generic feature for narrative writing, was assigned to this category.

**Table 6:** The axial coding of narration monitoring.

Open coding	Writing examples
Keep to past tense	This story happened in the past, so I should use past tense or past continuous tense.
Monitor the stylistic features of narrative writing	Everyday language is more suitable for story writing.
Imitate the source style	The words used in the source text were quite simple. So, I did not use complex words and expressions.
Use details	Adding details here would make the story impressive. *He put down…*
Monitor narration structure	*John* helped a thief…This is the beginning of everything. The continuation was the result.

#### 4.1.6. Continuity evaluation

Participants regarded the connection between the source and the continuation as a crucial aspect of continuation writing. Therefore, they placed significant importance on assessing the coherence and cohesion of their writing. As depicted in Table 7, EFL learners engaged in the practice of rereading their continuations to evaluate the flow of the story, ensuring local coherence. They also checked for the removal of any unresolved suspensions to achieve global coherence.

**Table 7:** The axial coding of evaluate continuity.

Open coding	Writing examples
Check global coherence	It seems that I have completely ignored the robbery so far…The robber should be caught…*All the robbers were…*
Reread the whole story	*John went as fast as a horse.* I used a really good metaphor here (to build up the source-continuation connection)
Check story completeness	The robber was caught, the protagonist cleared his name, and the money was found, so there is no more suspense in the story, and the story is complete.
Check the story flow	Maybe I had written too much on the running… How can I need to push the story forward and complete the task (within 150 words)?

### 4.2. Reading-writing connection

Table 8 shows five SCWT writing process factors identified in the PCA conducted prior to the CFA. These factors are ideational planning, skill integration, narration monitoring, reading monitoring, and continuity evaluation. Descriptive statistics reveal that the 411 questionnaire responses collected for the CFA study displayed a normal distribution, with all skewness and kurtosis values remaining within the range of −2 and + 2.

**Table 8:** Factor Information (*N* = 411).

Factor	Item (Original scale)/Reliability	*Mean*	SD	*Skewness*	*Kurtosis*
Ideational planning	1. Develop the story based on common logic (Planning)	3.98	0.88	−0.80	0.64
	2. Develop the story based on the source (Planning)	3.85	0.84	−0.44	−0.09
	3. Develop the continuation by inference making (Planning)	3.93	0.84	−0.67	0.58
Total	0.72	3.92			
Continuity evaluation	13. Check the story flow (Evaluate continuity)	3.17	0.99	0.06	−0.42
	14. Reread the whole story (Evaluate continuity)	3.47	1.20	−0.22	−1.00
	10. Check story completeness (Evaluate continuity)	3.79	0.89	−0.42	−0.23
Total	0.64	3.48			
Skill integration	5. Use source expressions directly (language monitoring)	2.94	1.01	0.30	−0.54
	8. Imitate the writing style of the source (Narration monitoring)	2.54	1.02	0.46	−0.15
	9. Select the appropriate storyline (Planning)	2.75	0.94	0.32	−0.12
Total	0.74	2.74			
Reading monitoring	4. Interpret the source based on inference-making (Comprehension monitoring)	3.58	0.93	−0.43	−0.03
	6. Look for key information and details (Source reading)	3.29	1.00	−0.19	−0.43
	7. Summarize the main ideas of the source (Comprehension monitoring)	3.45	1.00	−0.31	−0.40
Total	0.67	3.41			
Narration monitoring	11. Monitor the stylistic features of narrative writing (Narration monitoring)	3.56	1.12	−0.49	−0.50
	12. Integrate dialogs into the continuation (Narration monitoring)	3.28	0.91	0.00	−0.26
	15. Monitor the narration structure (Narration monitoring)	3.28	1.00	−0.03	−0.44
Total	0.64	3.37			
Total	0.87	3.39			

While the categories of narration monitoring and continuity evaluation remained mostly unchanged, significant changes were made to the grouping of items in the other three coding categories. Firstly, the axial coding category of planning was renamed ideational planning, as only the content planning item was retained after conducting the PCA. Secondly, the categories of source reading and comprehension monitoring were merged into a comprehensive factor named reading monitoring. Thirdly, the PCA identified a new factor consisting of one item from each of the three axial coding categories. These three items were related to narration monitoring, language monitoring, and planning. Since these items represented processes that involved the integration of skills (Knoch and Sitajalabhorn, 2013), this new factor was named accordingly.

The connections among the SCWT writing processes, as identified and analyzed through grounded theory, PCA, and CFA, are presented in Table 9 to demonstrate their relationship.

**Table 9:** SCWT process findings: from grounded analysis to factor analysis.

Axial coding categories	PCA findings	Factors included in the CFA model
1. Source reading	1. Reading monitoring (comprised of items originally from source reading and comprehension monitoring processes)	1.Reading monitoring
2. Comprehension monitoring		
3. Planning	2. Ideational planning (renamed because the only item related to content generation remained)	2.Ideational planning
4. Language monitoring	3. Skill integration (comprised of items originally from planning, language monitoring, and narration monitoring processes)	3. Skill integration
5. Narration monitoring	4. Narration monitoring	4.Narration monitoring
6. Continuity evaluation	5. Continuity evaluation	(Deleted due to large measurement error)

The initial CFA analyses revealed that the continuity evaluation factor had a large measurement error close to 1, and therefore, it was excluded from the model comparison study. The data fit indices presented in Table 10 indicated that the baseline CTF model was the best fit for connection among the remaining four factors (χ^2^/*df* ≤ 1.383, TLI ≥ 0.980, CFI ≥ 0.986, RMSEA≤0.031, and SRMR≤0.033).

**Table 10:** Fit indices for model comparisons.

Models	*χ* ^2^	*df*	*χ*^2^ */df*	*p*	TLI	CFI	RMSEA	SRMR
OTF	495.494	54	9.176	0.00	0.578	0.548	0.654	0.240
UTF	267.615	54	4.956	0.00	0.796	0.833	0.098	0.070
HTF	73.170	50	1.463	0.02	0.976	0.982	0.034	0.036
CTF	66.399	48	1.383	0.04	0.980	0.986	0.031	0.033
MCTF	66.409	49	1.355	0.05	0.982	0.986	0.029	0.033

Then, we conducted a further analysis to test a modified CTF (MCTF) model that explored the relationships among the four factors. Although the chi-square difference between the CTF and MCTF models was insignificant (Δ*χ*2 = 0.10, *df* = 1, *p* = 0.752), we preferred the MCTF model for two reasons. Firstly, it had a better model fit, as indicated by the probability level (*p* = 0.05) and TLI (0.982), CFI (0.986), and RMSEA (0.029) values. Secondly, it explicitly displayed the causal effects of reading and writing processes on the skill integration process, which addressed RQ 2.

In the CFA model, all standardized estimated loadings on the latent construct were higher than the threshold value of 0.50 (Raykov and Marcoulides, 2008). The model revealed that two monitoring processes associated with reading and writing skill use mediated the effects of ideational planning. However, only the reading monitoring process had direct and significant impacts (*β* = 0.67, *p* = 0.00), with the skill integration exerting a small and insignificant effect, on the factor of narration monitoring

## 5. Discussion

### 5.1. Writing process in the EFL integrated writing

**Figure 1:**
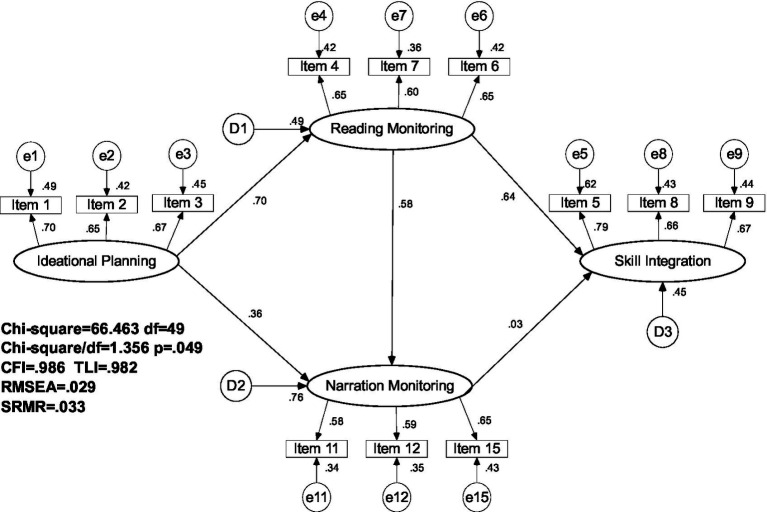
Relationships between writing processes in the EFL integrated writing task of SCWT.

We derived six main writing processes (see Figure 1), namely source reading, comprehension monitoring, planning, language monitoring, narration monitoring, and continuity evaluation, from the TAPs collected from fourteen learners. The presence of multiple monitoring processes highlighted the interplay between reading, writing, and language as fundamental skills supporting integrated EFL writing. Furthermore, the specific evaluation focused on maintaining continuity between the source and the continuation, showcasing the efforts made by EFL learners to adapt to the requirements of continuation writing. These contextual descriptions of the EFL writing process, derived from rich and detailed analyses of writing process data, align with the language-focused and problem-solving nature of EFL writing (Manchón et al., 2009).

In addition to identifying unique cognitive processes within the EFL context, this study also demonstrated that EFL learners engaged in an interactive and recursive composing process (Plakans, 2008; Yang, 2012). The detailed descriptions of axial coding schemes (refer to Section 4.1.1–6) revealed that the identified processes were not entirely independent of each other in sustaining SCWT writing. For example, source reading served as a foundational operation supporting comprehension monitoring, and these reading-related processes formed an essential prerequisite for ideational planning. In the process of constructing meaning for the continuation, learners engaged in rereading the entire story, including both the source and the continuation, to ensure and enhance overall continuity. These intensive interactions between reading and writing demonstrated a close connection between the two. In other words, EFL learners leveraged the complementary perspectives offered by reading and writing (Grabe and Zhang, 2016) to enhance source comprehension and facilitate continuation production.

### 5.2. Reading-writing connection in the EFL integrated writing

We employed the CFA approach to analyze 486 EFL SCWT questionnaire responses to validate and further examine qualitative findings in terms of process categories and their connections. The CFA model confirmed that most qualitative coding categories could be generalized to the larger context, as five out of six axial categories were retained after the PCA. After deleting the continuity evaluation factor, which had a large measurement error, we compared the fitness indices of hypothesized models to explore interactions among ideational planning (echoing the original category of planning), reading monitoring (echoing original categories of reading process and comprehension monitoring), narration monitoring, and the newly identified factor of skill integration (combining original categories of narration monitoring, language monitoring, and planning).

The final MCTF model selected to present the process interaction showed that EFL learners engaged in a meaning-making process driven by the ideational planning process. The overarching process directly and significantly influenced both reading and writing processes. The reading monitoring process then mediated the impacts of ideational planning on both reading monitoring and skill integration. That is, SCWT production processes were driven by both self-generated ideas (*β* = 0.36) and source text ideas (*β* = 0.56), confirming the previous conception that this is a content-responsible task requiring original ideas and source comprehension (Ye and Ren, 2023).

The larger regression weight of the monitor reading process on the skill integration could be explained in two ways. One was that the ideational planning process only provided conceptual support. The source text provided both conceptual and textual support for the production process (Ye and Ren, 2019), hence how learners read was a better predictor of how they wrote. Moreover, EFL writers might find it challenging to write and think simultaneously (Weigle, 2004). Therefore, although writers plan about the content before writing, they might not be able to execute the ideational planning during writing. Given the difficulty in putting one’s ideas into words reported in Table 4 and the range of language challenges reported in Table 5, it is understandable that participants relied more on source ideas, along with available linguistic expressions, in the production process. In addition to the greater impacts on the writing process, evidence of reading importance also lay in its exclusive direct and positive effects (*β* = 0.67) on skill integration. Taken together, the reading monitoring process, explained around 50–60% of variances in production-oriented processes including narration monitoring and skill integration process. While previous cognitive process (Yang and Plakans, 2012; Yang, 2014) and skill impacts (Wang and Qi, 2013; Liu and Chen, 2016) studies showed that writing and skill integration determined integrated task success, the current study showed that both factors were under the influence of reading. Hence, we believe more studies are needed to explore the importance of reading for integrated writing to expand the relatively small body of literature (Plakans, 2009b; Weigle et al., 2013).

Moreover, the current findings align with the research by Ye and Ren (2023) by illustrating the intricate interaction pattern among cognitive processes in EFL integrated writing. The present study, employing a self-rated process frequency questionnaire, corroborated the results obtained from a self-rated process importance questionnaire used in the aforementioned study (Ye and Ren, 2023) in a couple of ways. Firstly, it demonstrated the close connection between reading and skill integration processes, revealing that writers employed a reading-to-connect process to establish the link between reading and writing. Secondly, it highlighted the language-oriented nature of EFL integrated writing. The current study identified an axial coding scheme for language monitoring, thus supporting Ye and Ren (2023) in emphasizing the significance of fundamental language use. Therefore, we argue that the research scope of EFL integrated writing processes should be expanded to include writing, reading, and language-oriented processes in order to present a comprehensive view of the integrated writing process.

This study identified skill integration as a new factor in the cognitive processes of integrated writing. This composite factor encompassed both discourse synthesizing and source using processes, thus providing empirical support for the broad theoretical conception of the integrated writing construct (Knoch and Sitajalabhorn, 2013). Previous studies have demonstrated a strong connection between metacognitive processes, e.g., planning and monitoring, and the reading-to-writing dimensions, as observed in the discourse synthesizing process in Yang and Plakans (2012) and Yang (2014), as well as the reading-to-connect process in Ye and Ren (2023). However, the present study revealed that only reading monitoring had a direct and significant impact on skill integration. The impact of narration monitoring was direct but not significant, and the ideational planning process had no impact. We propose two possible explanations for this inconsistency. Firstly, although the grounded analysis identified the presence of a conventional planning process, only items related to content generation were included in the subsequent factor analysis. The change in the construct coverage within the planning process may account for the inconsistency. Secondly, the participants may have played a role. Since the SCWT was not yet included in the NMET (National Matriculation English Test) at the time of data collection, the participants had received varying degrees of instruction on the task prior to taking the test. This may explain why they failed to utilize skill integration to support the transformation of ideational planning.

## 6. Conclusion

To keep pace with global developments in integrated writing assessment practices, this mixed-methods study aimed to explore the cognitive processes undertaken by EFL writers and model the interaction pattern to demonstrate how they build the reading-writing connection. The findings revealed that reading played a central role in mediating the impact of ideational planning and directly influenced narration monitoring and skill integration processes.

Theoretically, this study supports the existence of a skill integration factor encompassing discourse synthesizing and source using processes, thereby endorsing the broad conception of the key integrated writing construct (Knoch and Sitajalabhorn, 2013). Additionally, it aligns with the proposal by Ye and Ren (2023) to develop a comprehensive understanding of the integrated writing process. Therefore, we suggest that future studies on EFL integrated writing processes should consider incorporating skill-oriented processes, such as writing, reading, and language use, to fully address contextual specificity. Finally, this study emphasizes the importance of reading in scaffolding production and skill integration processes, shedding light on this often overlooked aspect of integrated writing.

Methodologically, this study establishes a dialog between process studies and skill impact studies, which are the two main approaches for investigating the construct of integrated writing (Zhu et al., 2016). For the first time, empirical investigation was conducted on the connections between skill-oriented processes (e.g., reading and writing) and skill integration processes (e.g., discourse synthesizing and source using). Previous studies followed a confirmation paradigm and sought questionnaire evidence to validate L1-oriented conceptions, such as discourse synthesizing processes (Yang and Plakans, 2012; Yang, 2014; Shi et al., 2020; Liao et al., 2021). In contrast, this study took a different approach by employing a mixed-methods design and grounding all questionnaire items in EFL writer TAP data. We believe that this exploratory research design minimizes potential prompting effects from pre-conceptualized items, which could contaminate the findings and enhance the explanatory power of the results.

Pedagogically, EFL integrated writing test developers are advised to reexamine the underlying task construct and explore the inclusion of skill integration descriptors to achieve a balanced measurement of both writing and reading abilities. Furthermore, instructors are encouraged to enhance reading instruction to improve learners’ efficiency and effectiveness in discourse synthesis and source using.

The study has a few limitations, and therefore, the findings should be interpreted with caution. First, it was conducted prior to the release of the NMET in 2021, and the data was collected from freshmen, potentially impacting the generalizability of the findings to actual NMET performance. Second, this study primarily relied on self-reported data from EFL learners. Therefore, future studies will need to include other types of data, such as test scores and textual features, to help yield more valid, reliable, and insightful findings. Third, studies exploring other integrated writing tasks in EFL contexts would offer further evidence of the scaffolding use of reading, writing, and language use and highlight the importance of the reading process in supporting skill integration. Finally, the inconsistency in the categorization of writing processes could indicate limitations in the current data coding process. However, it could also suggest differences in perspectives between teachers and students regarding the integrated writing process, which should be further investigated.

## Data availability statement

The raw data supporting the conclusions of this article will be made available by the authors, without undue reservation.

## Author contributions

WY was responsible for data collection, statistical analysis, and manuscript writing. JL was responsible for the research design and manuscript revision. All authors contributed to the article and approved the submitted version.

## Funding

This paper is part of the work for the Talent Project of the Guangdong Polytechnic Normal University [2021SDKYB089], Guangdong Philosophy and Social Science Foundation Special Projects for Foreign Studies [GD23WZX01-04] and supported by the MOE Project of the Center for Linguistics and Applied Linguistics, Guangdong University of Foreign Studies [22JJD740019], Academic English Teaching Innovation for Value Education of Postgraduate Student [2022YJSP04008], and Innovative AI Translation Platform for Education-Industry Integration [22GPNUZDJS55].

## Conflict of interest

The authors declare that the research was conducted in the absence of any commercial or financial relationships that could be construed as a potential conflict of interest.

## Publisher’s note

All claims expressed in this article are solely those of the authors and do not necessarily represent those of their affiliated organizations, or those of the publisher, the editors and the reviewers. Any product that may be evaluated in this article, or claim that may be made by its manufacturer, is not guaranteed or endorsed by the publisher.
