# Supplementary material for: Exploring and modeling the reading-writing connection in EFL integrated writing
Source: Front Psychol. 2023 Jul 5;14:1161272. doi: 10.3389/fpsyg.2023.1161272 (PMC10355151; doi:10.3389/fpsyg.2023.1161272)
Supplement: Supplementary file 1 [file Data_Sheet_1.docx]

**Appendix 1 The SCWT writing prompt**

An unusual thing happened to John when he was on the way to work one day. As he walked along Park Avenue near the First National Bank, he heard the sound of someone trying to start a car. He tried again and again but couldn’t get the car moving. John turned and looked inside at the face of a young man who looked worried. John stopped and asked, “It looks like you’ve got a problem,” John said.

“I’m afraid so. I’m in a big hurry and I can’t start my car.”

“Is there something I can do to help? John asked. The young man looked at the two suitcases in the back seat and then said, “Thanks. If you’re sure it wouldn’t be too much trouble, you could help me get these suitcases into that taxi over there.”

“No trouble at all. I’d be glad to help.”

The young man got out and took one of the suitcases from the back seat. After placing it on the ground, he turned to get the other one. Just as John picked up the first suitcase and started walking, he heard the long loud noise of an alarm.

It was from the bank. There had been a robbery!

Park Avenue had been quiet a moment before. Now the air was filled with the sound of the alarm and the shouts of people running from all directions. Cars stopped and the passengers joined the crowd in front of the bank. People asked each other, “What happened?” But everyone had a different answer.

John, still carrying the suitcase, turned to look at the bank and walked right into the young woman in front of him.

She looked at the suitcase and then at him. John was surprised. “Why is she looking at me like that?” He thought. “The suitcase! She thinks I’m the bank robber!”

John looked around at the crowd of people. He became frightened, and without another thought, he started to run.

**Appendix 2 The initial SCWT questionnaire for PCA study**

|  | **Never** | **Seldom** | **Sometimes** | **Often** | **Always** |
| --- | --- | --- | --- | --- | --- |
| 1.Think about the writing requirement | 1 | 2 | 3 | 4 | 5 |
| 2.Read the source text carefully | 1 | 2 | 3 | 4 | 5 |
| 3.Underline the key information | 1 | 2 | 3 | 4 | 5 |
| 4.Plan the continuation globally before writing | 1 | 2 | 3 | 4 | 5 |
| 5.Use source expressions directly | 1 | 2 | 3 | 4 | 5 |
| 6.Develop the story based on common logic | 1 | 2 | 3 | 4 | 5 |
| 7. Develop the continuation based on background knowledge | 1 | 2 | 3 | 4 | 5 |
| 8.Develop the story based on the source | 1 | 2 | 3 | 4 | 5 |
| 9. Check the story flow | 1 | 2 | 3 | 4 | 5 |
| 10.Reread the source | 1 | 2 | 3 | 4 | 5 |
| 11.Plan the content and language during writing | 1 | 2 | 3 | 4 | 5 |
| 12.Imitate the writing style of the source | 1 | 2 | 3 | 4 | 5 |
| 13.Monitor the global coherence | 1 | 2 | 3 | 4 | 5 |
| 14.Select the most appropriate storyline | 1 | 2 | 3 | 4 | 5 |
| 15. Reread the continuation | 1 | 2 | 3 | 4 | 5 |
| 16.Consider the length | 1 | 2 | 3 | 4 | 5 |
| 17. Develop the continuation based on inference-making | 1 | 2 | 3 | 4 | 5 |
| 18. Check the story completeness | 1 | 2 | 3 | 4 | 5 |
| 19.Use diversified expressions | 1 | 2 | 3 | 4 | 5 |
| 20. Interpret the source based on inference-making | 1 | 2 | 3 | 4 | 5 |
| 21. Lexical use (spelling, plural forms, etc.) | 1 | 2 | 3 | 4 | 5 |
| 22. Look for key information and details | 1 | 2 | 3 | 4 | 5 |
| 23. Use details to present the continuation | 1 | 2 | 3 | 4 | 5 |
| 24. Follow the tense of the source | 1 | 2 | 3 | 4 | 5 |
| 25. Summarize the main ideas of the source | 1 | 2 | 3 | 4 | 5 |
| 26. Monitor the stylistic feature of narrative writing | 1 | 2 | 3 | 4 | 5 |
| 27. Use pronouns and conjunctions to establish coherence | 1 | 2 | 3 | 4 | 5 |
| 28. Integrating dialogues into the continuation | 1 | 2 | 3 | 4 | 5 |
| 29. Use collocations or structures correctly | 1 | 2 | 3 | 4 | 5 |
| 30. Use collocations or structures correctly | 1 | 2 | 3 | 4 | 5 |
| 31. Monitor language accuracy | 1 | 2 | 3 | 4 | 5 |
| 32. Monitor the narration structure | 1 | 2 | 3 | 4 | 5 |

**Appendix 3 The SCWT questionnaire for CFA study**

**This questionnaire is part of an academic study, and all the personal information and responses will only be used for the said study.**

|  | **Never** | **Seldom** | **Sometimes** | **Often** | **Always** |
| --- | --- | --- | --- | --- | --- |
| 1. Develop the story based on common logic | 1 | 2 | 3 | 4 | 5 |
| 1. Develop the story based on the source | 1 | 2 | 3 | 4 | 5 |
| 1. Develop the continuation by inference making | 1 | 2 | 3 | 4 | 5 |
| 1. Interpret the source based on inference-making | 1 | 2 | 3 | 4 | 5 |
| 1. Use source expressions directly | 1 | 2 | 3 | 4 | 5 |
| 1. Look for key information and details | 1 | 2 | 3 | 4 | 5 |
| 1. Summarize the main ideas of the source | 1 | 2 | 3 | 4 | 5 |
| 1. Imitate the writing style of the source | 1 | 2 | 3 | 4 | 5 |
| 1. Select the most appropriate storyline | 1 | 2 | 3 | 4 | 5 |
| 1. Check story completeness | 1 | 2 | 3 | 4 | 5 |
| 1. Monitor the stylistic features of narrative writing | 1 | 2 | 3 | 4 | 5 |
| 1. Integrate dialogues into the continuation | 1 | 2 | 3 | 4 | 5 |
| 1. Check the story flow | 1 | 2 | 3 | 4 | 5 |
| 1. Reread the continuation | 1 | 2 | 3 | 4 | 5 |
| 1. Monitor the narration structure | 1 | 2 | 3 | 4 | 5 |

Gender：□Male □Female Age：_______

Source familiarity: □Easy □Medium □ High

Task familiarity: □Easy □Medium □ High
